# Pulmonary artery sling in a symptomatic newborn

**DOI:** 10.34172/jcvtr.2021.07

**Published:** 2021-01-18

**Authors:** İlker Mercan, Muhammet Akyuz, Onur Işık

**Affiliations:** Department of Pediatric Heart Surgery, University of Health Sciences Tepecik Training and Research Hospital, Izmir, Turkey

**Keywords:** Pulmonary Arterial Sling, Airway Obstruction, Surgery

## Abstract

Pulmonary arterial sling (PAS) is a relatively rare congenital anomaly in which left pulmonary artery branch originates abnormally from the right pulmonary artery, eventually resulting with respiratory symptoms, due to airway obstruction. In this report, we present a PAS in a neonate who showed progressive respiratory distress in the second week following delivery. At 25 days of age, the patient underwent total surgical correction of the anomaly, during which left pulmonary artery reimplantation to main pulmonary artery without the use of cardiopulmonary bypass was employed. Following an uneventful recovery, the patient was discharged eighteen days after surgery.

## Introduction

Pulmonary artery sling is a rare type of vascular ring that causes compression in the distal trachea and right main bronchi.^1,2^ This malformation can be defined as the orientation of the left pulmonary artery originating from the posterior of the right pulmonary artery, going through the extrapericardial route and again passing through the posterior of the trachea and the anterior of the esophagus to the left hemithorax and from there to the hil of the left lung.^2^ As a result of this rare course anomaly, compression and related symptoms are seen in the right bronchial origin. If the ductus arteriosus between the main pulmonary artery and the aorta completes the ring together, the left main bronchus may also be affected.^2^ Respiratory symptoms of different severity may develop as a result of compression of the lower parts of the trachea and right and left bronchus. These symptoms can range from common wheezing to air trapping, recurrent pneumonia, persistent atelectasis, and dysphagia. In this article, we present a newborn patient who was admitted to the hospital due to respiratory distress and who developed a life-threatening severe clinical condition with the need for mechanical ventilation.

## Case Presentation

**Figure 1:**
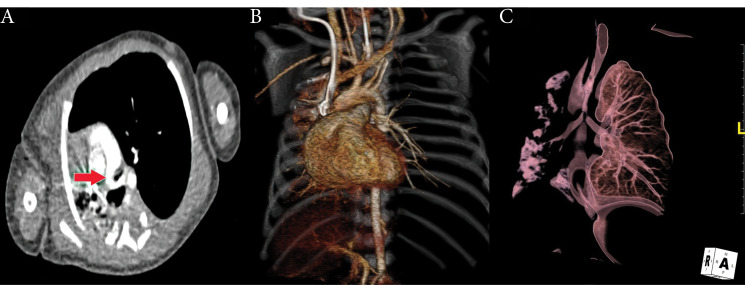
Axial section of CT showing aberran course of the left pulmonary artery and narrowing of the tracheal bifurcation (Red arrow). B. 3D CT image showing the left pulmonary artery that not branch out from the pulmonary trunk. C. 3D CT image showing the narrowing of the tracheal bifurcation

**Figure 2:**
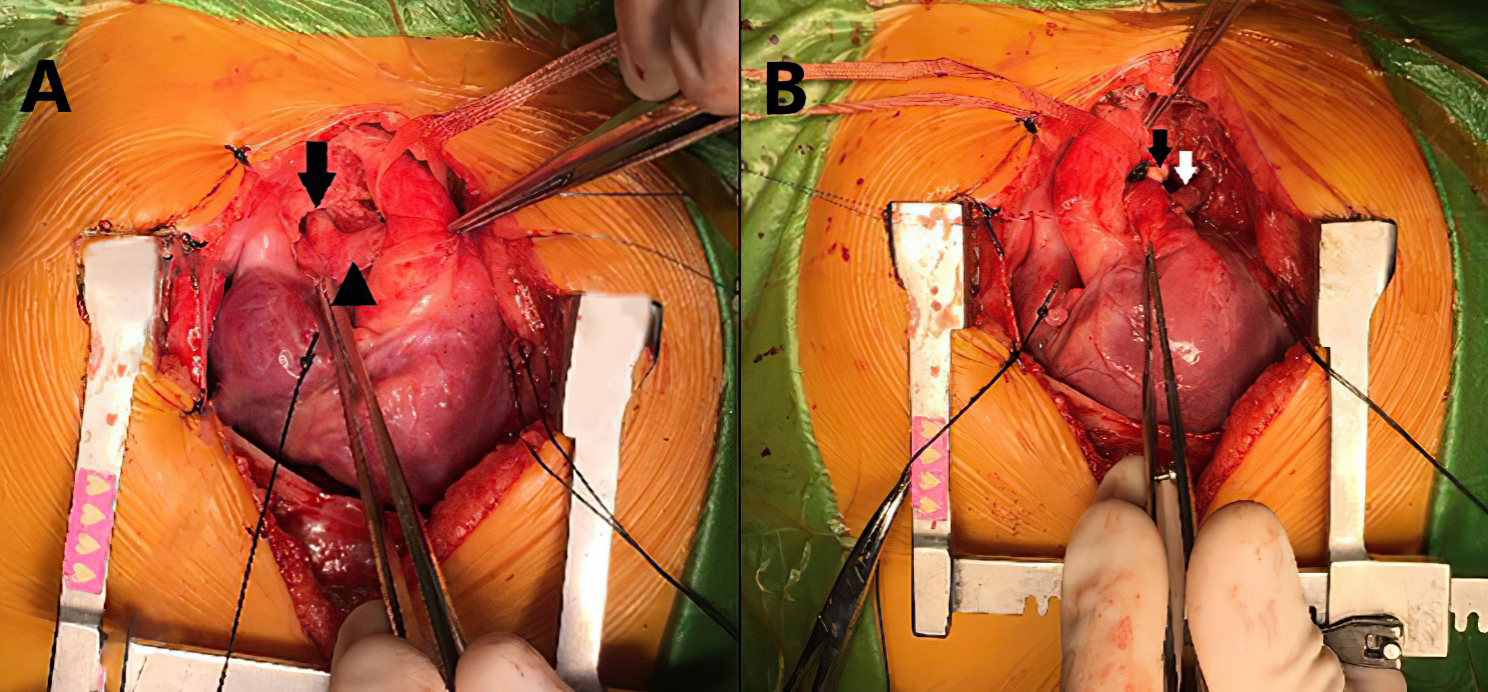
Intraoperative view of branching of the left PA(black arrow) from right PA (arrowhead). B. Intraoperative view of branching of the left PA from the pulmonary trunk after anastomosis (white arrow), Left PA stump zone (black arrow)

**Figure 3:**
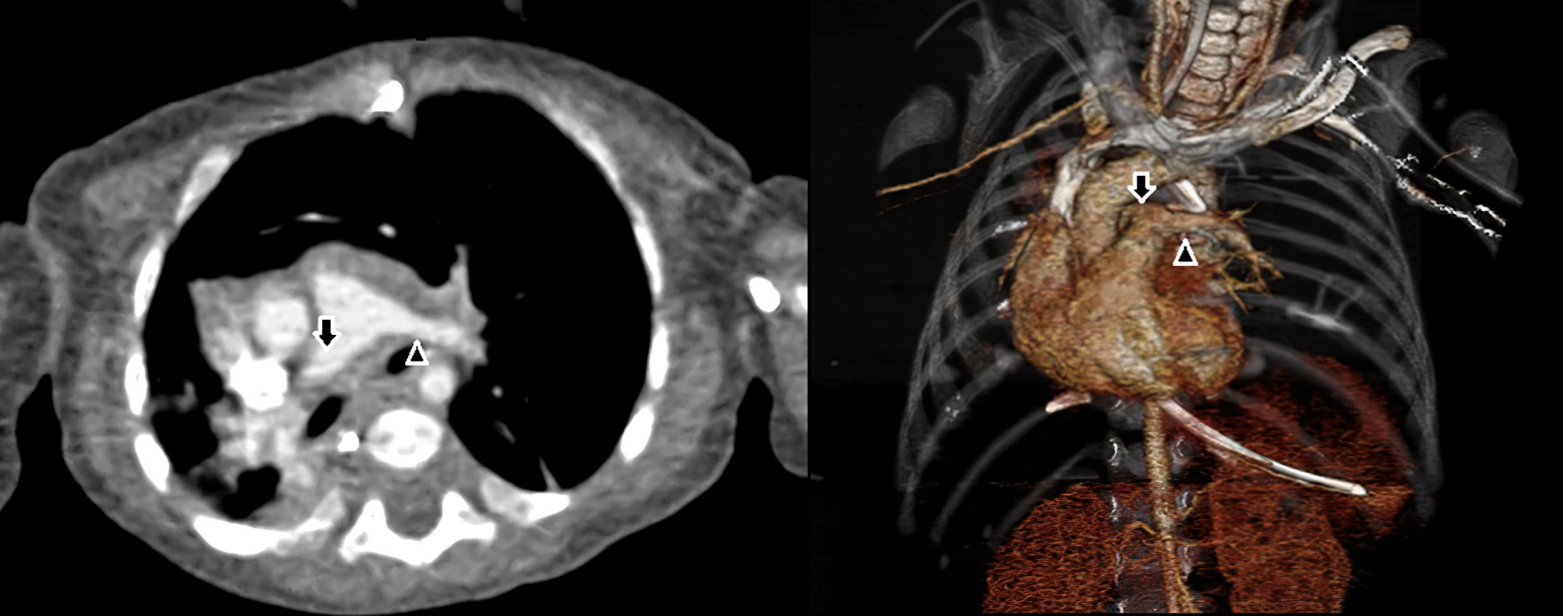
The postoperativecontrolCT images showing normal branches of the pulmonary trunk (arrowhead: left PA, black arrow: right PA).

The patient, who has had wheezing that has been ongoing since birth, was born at 37 weeks of age, weighing 2700 grams, and applied to our hospital emergency room due to respiratory distress. At the time of application, he was 45 days old and weighed 3700 grams. After the first assessment, pediatric intensive care hospitalization was made by intubation. It was evaluated by transthoracic echocardiography, but no intracardiac or extracardiac pathology was detected. On the chest radiograph, all segments of the right lung were atelectatic. And the emphysematous appearance in the left lung attracted attention. Therefore, a computed tomography (CT) examination was planned. In CT examination, it was observed that the left pulmonary artery originated from the posterior of the right pulmonary artery, and reached between the trachea and esophagus in the extrapericardial area and reached the hilus of the left lung (Figure 1). While the left pulmonary artery was originating from the right pulmonary artery, going to the right of the trachea and towards the posterior, it was pressing the left bronchus at the level of the bifurcation (Figure 1). Written consent of the family was obtained, and surgery was decided for the infant in urgent conditions. Median sternotomy was performed and mediastinum was reached. After the preparation of mediastinal structures, ligamentum arteriosum was divised and their stumps were closed primary. The right and left pulmonary arteries were dissected throughout the length and released. The abnormal course of the left pulmonary artery from the right pulmonary artery to the left was observed to the right of the midline (Figure 2a). Aortic and right atrial cannulation was performed. Normothermic cardiopulmonary bypass was initiated. The left pulmonary artery was divised. The right pulmonary artery primary was repaired. After releasing the left pulmonary artery, reanastamosis was performed at the appropriate localization on the main pulmonary artery (Figure 2b). The patient was followed up in the intensive care unit as intubated postoperatively and extubated on the 5th postoperative day. Postoperative atelectasis and prolonged intubation-associated pneumonia were other challenges we had to overcome. Non-invasive mechanical ventilation support was given for two days after extubation. Then, respiratory support was continued with a high flow nasal cannula. The patient was followed up in the intensive care unit for a total of 15 days. On the postoperative 15th day, she was taken to the clinical ward. In the ward, the patient was followed up with nasal oxygen support (2 liters / min) for two days. On the 17th postoperative day, she was followed up in the service at room air. The patient was discharged on the postoperative 18th day. The enoxaparin treatment, which was started on the postoperative day 0, was continued for 3 months (0.5 mg/kg/dose subcutaneously every 12 hours). In the follow-up of the patient, control pulmonary CT imaging performed 1 month after discharge revealed no stenosis in the anastomosis area of the left pulmonary artery (Figure 3). The patient has been followed up asymptomatically for 2 years after discharge.

## Discussion

Pulmonary artery sling, which has abnormal origination and continuity in the path where the left pulmonary artery originates from the right pulmonary artery, is a rare vascular anomaly. ^3,4^ It is frequently associated with other congenital heart diseases and pathologies related to tracheobronchial compression.^3^ Although the clinician’s suspicion and experience are important in diagnosis; It is usually possible to identify anomaly with a detailed echocardiographic examination. It can be noted as the first clue that classical pulmonary artery bifurcation cannot be seen on echocardiography. In addition, the importance of echocardiography (TTE) is indisputable in detecting intracardiac anomalies. However, after the diagnosis with TTE, additional imaging methods are needed to confirm this diagnosis. This additional imaging method may assist the need for bronchoscopy, barium esophagography, magnetic resonance imaging (MRI) and CT examinations. Bronchoscopy examination is useful in determining the location and extent of tracheal stenosis. However, MRI and CT examinations are valuable in terms of visualizing both vascular and other structures and making an accurate surgery plan. In such cases, our clinical preference is generally in favor of CT. Although MRI is avoided from the contrast material caused by tomography and the harmful effects of radiation, it may not be suitable for every patient because of the long duration of the examination, its being expensive and requiring sedation. In addition, CT imaging outputs are completed in a short time, and the radiation doses decreasing to low levels due to the advances in the technology of the devices are the prominent aspects of this examination.

After the diagnosis is clear, the patient’s condition-specific decisions should be made in the timing of surgery and in deciding on the procedure. Since PAS is an extremely rare malformation, its natural course in the neonatal period is not fully understood. Some authors reported that the diagnosis of the disease alone is an indication for surgical intervention.^5^ While an elective surgery plan can be made in the presence of mild and moderate symptoms, it may be necessary to make an operation plan without losing time in patients with mechanical ventilation needs due to respiratory problems. It was decided to operate in urgent conditions because our patient was followed up in the mechanical ventilator in the intensive care unit, he needed high pressure and oxygen support and had difficulty in managing blood gas values. We think that elective surgery planning can be done in cases with milder clinical conditions. In the planning of the surgery, how and where to make the anastomosis to the normal localization of the aberrant left pulmonary artery, evaluation of the tracheal branching and cannulation should be planned in order to correct the accompanying intracardiac anomalies. Median sternotomy incision and cardiopulmonary bypass (CPB) is the most suitable option for performing the specified procedures in a single session and safely. Left pulmonary artery division and reimplantation can be performed safely with CPB. At the beginning of this procedure, we think that the division of the ductus arteriosus both relieves tracheal compression and accelerates dissection and anastomosis.

The difference that differs from other ring types in the surgical correction planning of pulmonary artery sling anomaly is that some patients should also be treated with tracheal branching. In the study’s title on this subject, the anomaly has been named as ring-sling complex. The reason for this naming is; In addition to the left pulmonary artery aberrant course and tracheal compression in this course, tubular complete tracheal ring segments are absent in the tracheal branches without membranous section.^6^ It is very difficult to evaluate in which cases and by which procedure a surgical intervention should be performed on the tracheal branches. Although there is no definite idea about this in the literature; It seems that the idea that intervention is required in patients with a complete tracheal ring where the narrow segment is long and the lumen is very narrow.^7^ According to the studies conducted, we can say that morbidity and mortality increased significantly in patients requiring surgical intervention in tracheal branches.^4^ In the patient we presented, although the tracheal stenosis is serious, it is a short segment; After the division of the ductus arteriosus and the aberrant left pulmonary artery was released between the trachea and the esophagus to normal position, additional tracheal procedure was not considered due to the drop in airway pressures.

## Conclusion

Pulmonary artery sling is difficult to diagnose due to its rarity and a wide spectrum of symptoms. Although surgical treatment planning is easy, due to complicated cases diagnosed late, a problematic intensive care process occurs after surgery. Therefore, it is important to be diagnosed early. As a result, the surgical results of patients with this anomaly can be reduced by multidisciplinary teamwork.

## Competing interests

None.

## Ethical approval

None.
